# Occurrence of Apicomplexa protozoa in wild birds in the Northeast region of Brazil

**DOI:** 10.1590/S1984-29612023020

**Published:** 2023-04-17

**Authors:** Gabriela Hémylin Ferreira Moura, Gleyce Silva do Nascimento, Pollyanne Raysa Fernandes de Oliveira, Vanessa Silva Santana, Ayko Shimabukuro, Yuri Marinho Valença, Cecilia Calabuig, Renata Pimentel Bandeira de Melo, Rinaldo Aparecido Mota

**Affiliations:** 1 Laboratório de Doenças Infectocontagiosas dos Animais Domésticos, Departamento de Medicina Veterinária, Universidade Federal Rural de Pernambuco - UFRPE, Recife, PE, Brasil; 2 Laboratório de Ecologia e Conservação de Fauna Terrestre, Departamento de Biociência, Universidade Federal do Semi-Árido - UFERSA, Mossoró, RN, Brasil; 3 Centro de Triagem e Reabilitação de Animais Silvestres de Pernambuco - CETRAS-Tangara, Recife, PE, Brasil

**Keywords:** Toxoplasma gondii, *Sarcocystis* spp., *Isospora* spp., avifauna, Toxoplasma gondii, *Sarcocystis* spp., *Isospora* spp., avifauna

## Abstract

Protozoa of the Apicomplexa phylum are worldwide distributed with capacity to infect endothermic animals. The study of these protozoa in wild birds in Brazil is scarce. This study aimed to evaluate the occurrence of apicomplexan protozoa in wild birds in the Northeast of Brazil. From October to December 2019, brain tissue samples were collected from 71 captive birds from the Wild Animal Screening Center of the Pernambuco State (CETRAS-Tangara) and 25 free-living birds from the Caatinga biome in Rio Grande do Norte, totaling 96 animals (41 species). Brain fragments were subjected to molecular diagnosis by nested PCR for the 18s rDNA gene of Apicomplexa parasites, followed by DNA sequencing. This gene was detected in 25% (24/96) of the samples, and it was possible to perform DNA sequencing of 14 samples, confirming three genera: *Isospora*, *Sarcocystis* and *Toxoplasma* from eight bird species (*Amazona aestiva, Coereba flaveola, Egretta thula, Paroaria dominicana, Sporophila nigricollis, Cariama cristata, Columbina talpacoti, Crypturellus parvirostris*). The occurrence these coccidia in wild birds provides important epidemiological information for the adoption of preventive measures for its conservation. Future studies are needed to better understand the consequence of Apicomplexa infection in birds in Caatinga and Atlantic Forest biomes.

## Resumo

Protozoários do filo Apicomplexa são distribuídos mundialmente e com capacidade de infectar animais endotérmicos. O estudo destes protozoários, em aves silvestres do Brasil, é escasso. Objetivou-se avaliar a ocorrência de protozoários Apicomplexa em aves silvestres na região Nordeste do Brasil. De outubro a dezembro de 2019, foram coletadas amostras de encéfalo de 71 aves de cativeiro do Centro de Triagem e Reabilitação de Animais Silvestres de Pernambuco (CETRAS-Tangara). E 25 aves de vida livre do bioma Caatinga no Rio Grande do Norte, totalizando 96 animais (41 espécies). Os fragmentos de encéfalo foram submetidos ao diagnóstico molecular por *nested* PCR, para o gene 18s rDNA de protozoários Apicomplexa, seguido por sequenciamento do DNA. Este gene foi detectado em 25% (24/96) das amostras analisadas; foi possível realizar o sequenciamento de 14 amostras, confirmando-se três gêneros: *Isospora*, *Sarcocystis* e *Toxoplasma* em oito espécies de aves (*Amazona aestiva*, *Coereba flaveola*, *Egretta thula*, *Paroaria dominicana, Sporophila nigricollis, Cariama cristata, Columbina talpacoti*, *Crypturellus parvirostris*). A ocorrência destes coccídios nas aves silvestres fornece informações epidemiológicas importantes para a adoção de medidas preventivas tendo em vista sua conservação. Estudos futuros são necessários para melhor compreensão da consequência da infecção por Apicomplexa, em aves silvestres dos biomas Caatinga e Floresta Atlântica.

## Introduction

Apicomplexa phylum comprise intracellular obligate protozoa that are widely distributed worldwide and can infect a wide range of endothermic animals, including humans (Dubey, 2010). Members of this phylum have a complex biological cycle, with different infection stages, multiplying sexually and asexually (Besteiro, 2014). Among them, the genera *Cystoisospora, Eimeria, Isospora, Neospora, Sarcocystis*, and *Toxoplasma* are clinically and epidemiologically important for wild animals.

*Toxoplasma gondii* and *Sarcocystis* spp. are protozoa of the Sarcocystidae family most prevalent found in birds (Llano et al., 2022). Carnivorous birds can become infected with *Toxoplasma* by ingesting tissue cysts present in infected intermediate hosts, such as small rodents (Karakavuk et al., 2018), as well as birds can become infected by ingesting oocysts present in contaminated water, food, or soil (Lindsay et al., 2003). Studies carried out in domestic birds (*Gallus domesticus*), which are generally asymptomatic hosts, have reported a high prevalence of infection by *T. gondii* (Dubey et al., 2010; Magalhães et al., 2016). However, cases of acute toxoplasmosis have been reported in wild birds. In canary (*Serinus canaria*), severe clinical signs of encephalitis and ocular toxoplasmosis, including blindness, have been reported in Uruguay, Australia, Italy, New Zealand, and the United Kingdom (Dubey, 2002). These clinical signs are compatible with non-suppurative chorioretinitis in macrophages and tachyzoites of *T. gondii* in the subretinal space, in addition to tachyzoite aggregates in the retinal nerve fibers and cysts in the meninges and cerebellum (Williams et al., 2001). Moreover, fatal toxoplasmosis has been described in a vinaceous amazon parrot (*Amazona vinacea*) from Brazil (Ferreira et al., 2012) and in 12 little penguins (*Eudyptula minor*) from Australia (Campbell et al., 2022).

*Sarcocystis* spp. are cyst-forming Apicomplexan parasites in which the definitive hosts are predatory carnivores that become infected by ingesting tissue cysts (mature sarcocysts) present in the muscles of intermediate hosts, which, in turn, become infected by ingestion of water or food contaminated with sporocysts shed in the feces of the definitive hosts (Prakas & Butkauskas, 2012; Dubey et al., 2015). Birds can be definitive and/or intermediate hosts of several species of *Sarcocystis*, especially those that have potential pathogenicity. More than 25 *Sarcocystis* species, especially *S. falcatula* and *S. calchasi* are capable of forming cysts in the muscle tissue of birds, which can be highly pathogenic for Passeriformes, Psittaciformes, and Columbiformes (Kutkienė et al., 2012; Dubey et al., 2015).

Protozoa of the genus *Isospora* (Eimeriidae family) usually parasitize the enterocytes of birds and are responsible for enteric diseases. Furthermore, this protozoan may have an extra intestinal cycle and can be transported intracellularly through the blood stream to multiple organs, leading to the systemic manifestation of the disease (Sánchez-Cordón et al., 2007; Cushing et al., 2011), which is known as systemic isosporosis or atoxoplasmosis. Transmission often occurs via the fecal-oral route and is characterized by a direct life cycle. Clinical diseases are associated with stressful situations, immunosuppression, and young birds (Barbón et al., 2019). The parasite asexual reproduction occurs in intestinal and blood-circulating cells (lymphocytes and monocytes). The asexual stage of this parasite has been found in several somatic tissues, mainly liver, spleen, and intestine, and have been also detected in lungs, heart, pancreas, kidneys, proventriculus, gizzard, gonads, and brain (Sánchez-Cordón et al., 2007; Cushing et al., 2011; Mohr et al., 2017; Oliveira et al., 2018). Pathogenesis of systemic isosporosis and its etiologic agent have not been well characterized (Oliveira et al., 2018), however, taxonomically *Atoxoplasma* is considered a junior objective synonym of *Isospora* causing systemic disease. According to Barta et al. (2005), the genus concept for *Isospora* Schneider, 1881 applies to all the species that belong to the large clade of isosporoid coccidia infecting passerines and this nomenclature should be used instead of *Atoxoplasma* (Barta et al., 2005).

Studies on the occurrence of apicomplexan protozoa in wild birds in Northeast Brazil are important because of their impact on the imbalance of biomes in this region (Alves et al., 2013), as infections may cause disease and even death, which is a threatened to endemic species. These biomes (Caatinga and Atlantic Forest) have more than 800 endemic species, of which ~45% of Atlantic Forest species are considered threatened and may become extinct, because of trafficking and deforestation, which, associated with disorderly urbanization, facilitate the establishment of new interactions between hosts and parasites (Ribon et al., 2003; Hasui et al., 2018).

Given the scarcity of data on the occurrence of those protozoa in wild animals in the Northeast region of Brazil, the objective of this study was to identify Apicomplexa coccidia in wild birds from a screening center for wild animals and free-living birds.

## Material and Methods

### Sampling

Sampling was carried out between October and December 2019. Brain tissue samples from 71 wild birds of 27 species were collected from the Wild Animal Screening Center of the Pernambuco State (CETRAS-Tangara). These birds were found dead in the enclosures and submitted to necropsy to collect the brain fragments. This center houses hundreds of wild birds of different species, coming from voluntary delivery, seizure of trafficking, and open fairs by inspection agents. Additionally, 26 samples of wild free-ranging birds of 14 species were collected from the semi-arid region of the Caatinga biome in the state of Rio Grande do Norte. Information on the animals analyzed in this study is shown in Table 1.

**Table 1:** Taxonomic order, common name, scientific name, number of samples, and origin (municipality and state) of the wild birds analyzed in this study.

**Order**	**Common Name**	**Scientific Name**	**Number of samples**	**Origin**
Caprimulgiformes	Little Nightjar	*Hydropsalis parvula*	1	Mossoró / Rio Grande do Norte
Cariamiformes	Red-legged Seriema	*Cariama cristata*	7	Pendências / Rio Grande do Norte
Columbiformes	Picui Dove	*Columbina picui*	4	Mossoró / Rio Grande do Norte (urban área)
	Ruddy Ground-dove	*Columbina talpacoti*	1	Mossoró / Rio Grande do Norte (urban área)
	Plain-breasted Ground-dove	*Columbina minuta*	1	Alagoinha / Rio Grande do Norte
Cuculiformes	Smooth-billed Ani	*Crotophaga ani*	1	Mossoró / Rio Grande do Norte
	Coccyzus melacoryphus	*Coccyzus melacoryphos*	1	Mossoró / Rio Grande do Norte
Gruiformes	Purple Gallinule	*Porphyrio martinicus*	1	Pernambuco
Passeriformes	Great Kiskadee	*Pitangus sulphuratus*	4	Pernambuco
	Cinnamon Tanager	*Schistochlamys ruficapillus*	1	Pernambuco
	Double-collared Seedeater	*Sporophila caerulescens*	1	Pernambuco
	White-naped Jay	*Cyanocorax cyanopogon*	1	Mossoró / Rio Grande do Norte
	Double-collared Seedeater	*Sporophila caerulescens*	1	Mossoró / Rio Grande do Norte
	Chopi Blackbird	*Gnorimopsar chopi*	3	Pernambuco
	Red-cowled Cardinal	*Paroaria dominicana*	13	Pernambuco
			3	Mossoró / Rio Grande do Norte
	Violaceous Euphonia	*Euphonia violacea*	1	Pernambuco
	Yellow-bellied Seedeater	*Sporophila nigricollis*	7	Pernambuco
	Plumbeous Seedeater	*Sporophila plumbea*	1	Pernambuco
	Rufous-bellied Thrush	*Turdus rufiventris*	2	Pernambuco
	Sayaca Tanager	*Tangara sayaca*	2	Pernambuco
	Rufous-bellied Thrush	*Turdus rufiventris*	1	Rafael Fernandes / Rio Grande do Norte
	Tangara sayaca	*Tangara sayaca*	1	Rafael Fernandes / Rio Grande do Norte
	Bananaquit Coereba	*Coereba flaveola*	5	Pernambuco
	Blue-black Grassquit	*Volatinia jacarina*	2	Pernambuco
	Buff-throated Saltator	*Saltator maximus*	3	Pernambuco
Pelecaniformes	Snowy Egret	*Egretta thula*	1	Pernambuco
Piciformes	Green-barred Woodpecker	*Colaptes melanochloros*	3	Pernambuco
Procellariiformes	Cory's Shearwater	*Calonectris borealis*	1	Pernambuco
Psittaciformes	Cactus Parakeet	*Eupsittula cactorum*	1	Pernambuco
	Cactus Parakeet	*Eupsittula cactorum*	1	Pendências / Rio Grande do Norte
	Northern Red-shouldered Macaw	*Diopsittaca nobilis*	1	Pernambuco
	Scaly-headed Parrot	*Pionus maximiliani*	1	Pernambuco
	Orange-winged Amazon	*Amazona amazonica*	1	Pernambuco
	Southern Mealy Amazon	*Amazona farinosa*	1	Pernambuco
	Turquoise-fronted Amazon	*Amazona aestiva*	12	Pernambuco
Strigiformes	Ferruginous Pygmy-owl	*Glaucidium brasilianum*	1	Pernambuco
	American Barn Owl	*Tyto furcata*	1	Pernambuco
	Tropical Screech-owl	*Megascops choliba*	1	Pernambuco
Tinamiformes	Small-billed Tinamou	*Crypturellus parvirostris*	2	Pendências / Rio Grande do Norte

### DNA extraction and nested PCR

The brain fragments were macerated, weighed (25 mg), and subjected to DNA extraction using a commercial Wizard Genomic DNA Purification kit (Promega®). In order to evaluate contamination, a control sample (ultrapure water) was used in each round of DNA extraction. After extraction, the samples (including the controls of DNA extraction) were amplified by nested polymerase chain reaction (PCR) for the 18s gene of the ribosomal DNA (18S rDNA) of Apicomplexa protozoa, using 2µM of the external primers Tg18s48F (5'-CCATGCATGTCTAAGTATAAGC-3') and Tg18s359R (5'-GTTACCCGTCACTGCCAC-3'). Thermal profile consisted of an initial denaturation at 94 °C for 2 min, 30 cycles of denaturation at 94 °C for 30 s, annealing at 57 °C for 30 s, extension at 72 °C for 30 s, followed by a final extension at 72 °C for 1 min. It was followed by the nested PCR amplification using 2µM of the internal primers Tg18s58F (5'-CTAAGTATAAGCTTTTATACGGC-3') and Tg18s348R (5'-TGCCACGGTAGTCCAATAC-3'), according to the protocol described by Su et al. (2010), and Melo et al. (2020). The thermal profile was similar to the first PCR reaction, except for an increase to 35 cycles. Ultrapure water and the *Toxoplasma gondii* strain ME-49 were used as negative and positive control, respectively. Nested PCR products were visualized by electrophoresis in a 2% agarose gel stained with Blue Green (LGC®) and photodocumented.

Gel bands from the nested PCR products positively labeled for the 18S rDNA gene were purified using the QIAquick Gel Extraction Purification Kit (Qiagen), according to the manufacturer's protocol. The products were sequenced bidirectionally using the BigDye Terminator v3.1 Cycle Sequencing kit (Applied Biosystems). The oligonucleotides used for sequencing were the same as those for the second amplification step (Tg18s58F and Tg18s348R). Sequencing was performed by capillary electrophoretic separation on an ABI 3500 Genetic Analyzer sequencer (Applied Biosystems).

Sequencing data were collected using the Data Collection software (Applied Biosystems), and quality inspection was performed using Sequencing Analysis Software (Applied Biosystems). The sequences were analyzed by Staden Package 4.1.4 (Gene Codes Corporation, USA) and compared with the GenBank database using Basic Alignment Search Tools at the National Center for Biotechnology Information (www.ncbi.nlm.nih.gov) to identify genera or species.

## Results

Protozoan DNA of the phylum Apicomplexa was detected in 26.7% (19/71) of the brain samples from the birds collected at CETRAS in Pernambuco. Regarding free-ranging birds, 23.75% (5/25) tested positive. Control samples of DNA extraction were all negative on PCR. It was not possible to obtain sufficient concentration of amplified DNA for sequencing for all the PCR-positive samples. Thus, 14 samples were sequenced: nine samples from CETRAS birds and five samples from free-living birds, from which three genera were identified: *Isospora*, *Sarcocystis*, and *Toxoplasma* (Table 2).

**Table 2:** Identification of Apicomplexa phylum protozoan by nested Polymerase Chain Reaction (PCR) and DNA sequencing in brain fragment samples from 14 wild birds from Northeast Brazil.

**Common Name**	**Species**	**Number of Samples**	**Protozoan**	**Origin**
Red-legged Seriema	*Cariama cristata*	3	*Toxoplasma gondii*	Rio Grande do Norte
Ruddy Ground-dove	*Columbina talpacoti*	1	*T. gondii*	Rio Grande do Norte
Red-cowled Cardinal	*Paroaria dominicana*	2	*T. gondii*	Pernambuco
		1	*Isospora* spp.	Pernambuco
White-naped Jay	*Cyanocorax cyanopogon*	1	*T. gondii*	Rio Grande do Norte
Yellow-bellied Seedeater	*Sporophila nigricollis*	2	*T. gondii*	Pernambuco
Bananaquit *Coereba*	*Coereba flaveola*	1	*Sarcocystis* spp.	Pernambuco
Snowy Egret	*Egretta thula*	1	*T. gondii*	Pernambuco
Turquoise-fronted Amazon	*Amazona aestiva*	2	*T. gondii*	Pernambuco

Sequencing analysis of 12 positive samples indicated similarity of 100% with *T. gondii* sequences of the 18S rDNA gene (reference numbers from MT634248 to MT634250). One positive sample showed 99,6% similarity with *Isospora* sp. (reference numbers MW667591.1 and MW649139.1) and another positive sample was 100% similarity to *Sarcocystis speeri*, *Sarcocystis neurona*, and *Sarcocystis felis* (reference numbers KP681854.1, KT207459.1, AH009986.2, AY656815.1).

*Toxoplasma gondii* was the most frequently identified protozoan in the birds of CETRAS-Tangara, detected in two Red-cowled Cardinal (*Paroaria dominicana*), Yellow-bellied Seedeater (*Sporophila nigricollis*), two Turquoise-fronted Amazon (*Amazona aestiva*), and one Snowy Egret (*Egretta thula*). *Isospora* sp. was also identified in a cockerel and *Sarcocystis* sp. in a bananaquit, which is the first report of this genus in the species *Coereba flaveola* in Brazil.

Among free-ranging birds from the Caatinga biome in the state of Rio Grande do Norte, *T. gondii* was the only protozoan identified in a Ruddy Ground-dove (*Columbina talpacoti*), White-naped Jay (*Cyanocorax cyanopogon*), and three Red-legged Seriema (*Cariama cristata*).

## Discussion

We detected Apicomplexa DNA in 25% (24/96) of the analyzed samples, and identified *T. gondii* (12.5%; 12/96), *Sarcocystis* spp. (1.04%; 1/96), and *Isospora* spp. (1.04%; 1/96) in the brain fragments of wild birds from the Northeast region of Brazil. Concentration and quality of the obtained amplified DNA limited the genetic sequencing of all positive samples, as they were directly collected from animal tissues where the parasites are heterogeneously distributed. Dubey (2010) also reported that PCR was effective for diagnosis, but less so for genotyping, owing to the small amount of genetic material found in the tissues.

In Brazil, studies on the prevalence and distribution of protozoa of the Sarcocystidae family in wild animals by the detection of the 18S rDNA gene are limited. Melo et al. (2020) detected *T. gondii* in 17.9% (7/39) of heart samples from marmosets (*Callithrix jacchus*) in the state of Pernambuco. The genera *Toxoplasma* (22.7%, 10/44) and *Sarcocystis* (11.4%, 5/44) were reported in wild free-ranging birds rescued in the state of Minas Gerais (Rêgo et al., 2021). Another study has shown 4.7% (91/1921) of bats with tissues positive for the 18S rDNA gene were detected, and eight genera of protozoa of the Sarcocystidae family (*Besnoitia, Cystoisospora, Hammondia, Hyaloklossia, Neospora, Nephroisospora, Sarcocystis*, and *Toxoplasma*) were identified in 50 samples of 16 bat species (belonging to the families Phyllostomidae, Molossidae, and Vespertilionidae) in the state of São Paulo (Cabral et al., 2021). In addition, Ludwig et al. (2021) detected the 18S rDNA gene in brain and heart tissue from six brown howler monkey (*Alouatta guariba clamitans*) and identified the genus *Sarcocystis* (4/6) and *Toxoplasma* (2/6).

Silva et al. (2018) isolated and genotypically characterized *T. gondii* from wild free-ranging or captive birds in the state of Pernambuco, Brazil, and described a non-virulent isolate of *T. gondii* in a free-living green-backed heron (*Butorides striata*). They also identified positive PCR samples for *T. gondii* in a spectacled owl (*Pulsatrix perspicillata*), an American kestrel (*Falco sparverius*), and a green-backed heron (*B. striata*), and warned of the risk of infection in endangered wild animals.

The identification of *T. gondii* in captive birds is a warning for wild animal conservation centers to take necessary measures, as severe clinical respiratory signs, blindness and encephalitis can occur in passerines with toxoplasmosis (Dubey, 2022). Factors such as capture stress, environment and food changes, immunosuppression, inadequate enclosures, and the presence of individuals from different locations favor the spread of infectious agents (Andrade et al., 2016; Silva et al., 2018). CETRAS receives and shelters several species of birds from trafficking of wild animals from the Caatinga and Atlantic Forest, which are illegally captured and kept in unfavorable conditions, such as mistreatment, food stress, and overpopulation, making them susceptible to diseases.

In the present study, Psittaciformes and Passeriformes had the highest number (62/71) of analyzed individuals from CETRAS-Tangara, as these birds are trafficked the most in Brazil. *P. dominicana* is the most illegally traded species in Recife, Pernambuco (Pereira & Brito, 2005), especially in the Northeast region of Brazil (Alves et al., 2013), as the region has a high biodiversity, along with socioeconomic problems that drive the illegal capture and sale of animals, in addition to the cultural issues of hunting and domestic breeding. In this study, fragments of brain tissue from 13 specimens *P. dominicana* from Pernambuco state were analyzed and two samples were positive for *T. gondii* DNA and in one sample the DNA of *Isospora* was detected.

The free-living birds of the Caatinga biome that were found positive for *T. gondii* in our study have different feeding habits, such as the crow, which is omnivorous, and eats arthropods, insects and fruits, small rodents, small snakes, fish, and other smaller birds. The red-legged seriema eats insects to small vertebrates, such as rodents, reptiles, amphibians, and other bird species, whereas the Ruddy Ground-dove eats grains from the ground. It is not possible to state how the animals became infected. However, a possible source of infection for free-living birds is soil or water contaminated with *T. gondii* oocysts (Lindsay et al., 2003), which is considered the most important form of *T. gondii* infection for animals (Dubey, 2010). In addition, there are cataloged wild felids in this biome, such as the Northern Tiger Cat (*Leopardus tigrinus*), Ocelot (*Leopardus pardalis*), Jaguarundi (*Herpailurus yagouaroundi*), Puma (*Puma concolor*) (Marinho et al., 2018) and Jaguar (*Panthera onca*) (Machado, 2020), which serve as definitive hosts of the parasite and may shed oocysts in feces contaminating the environment (Dubey, 2010). Another possibility is the ingestion of tissue cysts from infected small mammals and birds. Wild birds, intermediate hosts of *T. gondii*, can be considered bioindicators of the presence of this coccidia, and the detection of seropositive birds suggests that they have ingested sporulated oocysts from the soil, water, or contaminated food (Gondim et al., 2010), as well as the detection of protozoa DNA in animal tissues confirms the infection. Studies like ours can be performed without collecting blood to obtain sera and are a useful way to access environmental contamination with oocysts, since different avian populations feed directly on the ground (Dubey, 2002).

Caatinga is a unique Brazilian biome that is home to 182 species of endangered animals, of which 46 are endemic to this biome. This is mainly due to the expansion of agricultural activities that have already devastated >50% of the native vegetation. Thus, studying infectious agents, such as those of the Apicomplexa phylum, which have a negative impact on animal health, provides important data that help in the conservation of threatened species (Figueiredo, 2019).

The detection of *Isospora* sp. in brain tissue of CETRAS animal poses health risks to wild birds. This parasite is considered as endemic in wild birds, and infection does not result in a significant mortality rate, however cases of fatal disease may occur under the influence of stress, concurrent infections, or immunosuppression, especially for young birds in captivity (Cushing et al., 2011). *Isospora* extraintestinal infection has been reported in a wide variety of birds, such as canaries (Quiroga et al., 2000), blue-crowned laughingthrush (Jamriška et al., 2013; Mohr et al., 2017; Barbón et al., 2019), tanagers (Adkesson et al., 2005), green-winged saltator (Oliveira et al., 2018), sparrows, finches, starlings, and thrushes (Cushing et al., 2011). Clinical signs of atoxoplasmosis (systemic isosporosis) in birds are nonspecific and may include anorexia, diarrhea, ruffled feathers, reduction of pectoral musculature, distended abdomen, retracted neck, and respiratory difficulties (Maslin & Latimer, 2009).

Furthermore, we identified *Sarcocystis spp*. in wild captive birds. This protozoan has already been described in wild free-ranging birds in Brazil (Rêgo et al., 2021), especially *Sarcocystis falcatula*, which has already been associated with the mortality of 38 parrots in Parque das Aves in Foz do Iguaçu, Paraná (Godoy et al., 2009), with meningoencephalitis, and pulmonary alterations (Godoy et al., 2009; Dubey, 2010). Recently, Adão et al. (2021) reported sudden illness and death in a gray parrot (*Psittacus erithacus*) that lived with its owner and had contact with other birds. At necropsy, macroscopic and microscopic features confirmed the diagnosis of *S. falcatula.*

Although *Sarcocystis* infection is usually asymptomatic, studies have warned about the presence of this pathogen in free-ranging and captive birds as an important epidemiological indicator of environmental imbalance, in addition to highlighting the risks for species of the orders Psittaciformes, Columbiformes, and Passeriformes, which are most prone to trafficking and extinction risks. Although studies on *S. falcatula* and *S. calchasi* in wild birds are scarce, *S. falcatula* infections generally show hyperacute symptoms with severe lung injury, whereas *S. calchasi* can lead to meningoencephalitis (Dubey et al., 2015).

It is not possible to determine the moment at which the Apicomplexa infected the CETRAS birds, which may have occurred naturally or after the birds arrived at the enclosures. It is also not possible to comment on the infection sources, but it is known that the feeding habits of each species can influence the risk of infection. The species of birds that were positive for apicomplexan protozoans in this study had different feeding habits in nature.

The animals of the Psittacidae family, represented by the Turquoise-fronted Amazon (*Amazona aestiva*) and the Northern-red shouldered Macaw (*Diopsittaca nobilis*), feed on seeds and fruits, and foraging is done on tree branches. In restricted environments, these individuals remain in cages or nurseries and usually forage on the ground, which is the possible site of oocyst ingestion. The passerine diet consists of grains, seeds, fruits, and insects (Sick, 1997). In nature, due to shrub/tree feeding, oocyst ingestion is minimized, whereas in nurseries, foraging is carried out in feeders and on the ground, which increases the risk of oocyst ingestion. The Snowy Egret (*Egretta thula*) is an aquatic bird that lives near lakes and rivers and feeds on fish; therefore, infection is associated with pollution and improper dumping of contaminants that carry oocysts (Gwynne et al., 2010). In captivity, their diet is usually similar to that in the wild, especially fish, and the risk of infection is related to the quality of food.

## Conclusion

This is the most comprehensive study on the occurrence of Apicomplexa protozoa in wild birds from a rehabilitation center and in the wild in the Northeast region of Brazil. The results obtained confirms the infection of wild birds by apicomplexan coccidia (*Toxoplasma gondii*, *Isospora* sp. and *Sarcocystis* sp.) and provide important epidemiological information for the adoption of preventive measures for the conservation of wild birds in captivity and the environment.
